# Increased Functional Foods’ Consumption and Mediterranean Diet Adherence May Have a Protective Effect in the Appearance of Gastrointestinal Diseases: A Case–Control Study

**DOI:** 10.3390/medicines6020050

**Published:** 2019-04-09

**Authors:** Ioannis-Nektarios Elmaliklis, Athanasia Liveri, Basileios Ntelis, Konstantina Paraskeva, Ioannis Goulis, Antonios E. Koutelidakis

**Affiliations:** 1 Department of Food Science and Nutrition, University of the Aegean, Mitropoliti Ioakeim 2, 81400 Myrina, Limnos, Greece; ioannis.elmaliklis@gmail.com; 2 Department of Statistics and Insurance Science, University of Piraeus, Karaoli & Dimitriou 80, 18534 Piraeus, Greece; syn8ia@live.com; 3 G. Genimmatas Hospital, Leoforos Mesogeion 154, 11527 Athens, Greece; basildelis@yahoo.gr; 4 Konstantopoulio General Hospital, Agias Olgas 3, Nea Ionia, 14233 Athens, Greece; konparaskeva@gmail.com; 5 Ippokrateio Hospital of Thessaloniki, Konstantinoupoleos 49, 54642 Thessaloniki, Greece; hepatodpath@gmail.com

**Keywords:** functional foods, Mediterranean diet, gastrointestinal diseases, case–control study

## Abstract

**Background:** Epidemiological studies have suggested a possible correlation between nutritional factors and gastrointestinal diseases. **Methods:** A case–control study was designed in order to investigate if functional foods consumption and Mediterranean diet adherence have a positive effect in ulcerative colitis, Crohn’s disease, irritable bowel syndrome, and gastroesophageal reflux disease. In total, 142 patients (cases) and 147 gender-matched healthy people (controls) participated in the study. Functional food consumption was screened by using a Food Frequency Questionnaire based on the NHANES study, while Mediterranean diet adoption was evaluated by a 14-item Med Diet Assessment tool based on the PREDIMED study. The statistical analysis was performed with SPSS-22. **Results:** In the previous 2–3 years, the controls had more frequently consumed some categories (probiotics, prebiotics-enriched, and low-fat foods) and some kinds of functional foods (mountain tea, berries, pomegranate, oats, mastics, turmeric, soybeans, and raisins) compared to the cases (*p* < 0.05). Healthy people were more adherent to the Mediterranean diet than patients (*p* < 0.05). A multifactor analysis showed that the augmented score of the Mediterranean diet and the augmented consumption of categories and kinds of functional foods were protective factors in the appearance of gastrointestinal diseases. **Conclusions:** More studies should be conducted in order to further investigate the possible association between specific food components and gastrointestinal diseases’ pathophysiology.

## 1. Introduction

Gastrointestinal diseases, especially inflammatory bowel disease (IBDs), Crohn’s disease (CD,) and ulcerative colitis (UC), are chronic inflammatory conditions, which have been increasing in incidents, prevalence, and severity, in recent years. While there is genetic susceptibility to IBD, the probability of disease development is affected by diet, lifestyle, and endogenous factors, including the gut microbiota. For example, high intakes of mono- and disaccharides and total fats consistently increase the risk of developing IBD. A high vegetable intake may reduce the risk of UC, whereas increased fruit and/or dietary fiber intake possibly protects against CD. Both probiotics and prebiotics may modulate the gut microflora and reduce the likelihood of IBD regression. Dietary patterns and whole diet may be even more important to disease susceptibility than the levels of individual foods or nutrients [1].

Irritable bowel syndrome (IBS) is a common and poorly understood chronic condition that is treated with a great variety of drugs, bulking agents, dietary factors, and forms of psychotherapy, without notable enduring success [2]. Gastro-esophageal reflux disease (GERD) is a condition with a wide range of clinical manifestations. Population-based cross-sectional studies conducted worldwide suggest that up to 20% of the general population experiences heartburn and/or regurgitation at least weekly. The risk factors associated with GERD are overweight, consumption of alcohol, smoking, and having a family history of upper gastrointestinal symptoms [3,4,5,6,7,8]. The pathognomonic symptoms of GERD include heartburn or pyrosis, regurgitation, and dysphagia in the late stages of the disease. The etiology of GERD is largely unknown, while genetic factors perhaps play a crucial role [9,10,11].

In recent years, the concept of foods specifically developed to reduce the risk of diseases was introduced, aiming at health promotion. Functional foods are considered those foods, fresh or processed, that are intended to be consumed as part of the normal diet and contain biologically active components which offer the potential of enhanced health or reduced risk of diseases. Examples of functional foods include foods that contain natural or added specific minerals, vitamins, fatty acids, or dietary fibers, biologically active substances such as phytochemicals, antioxidants, and probiotics, the latter containing live beneficial microbial cultures [12,13,14,15,16,17]. Although many studies showed that dietary habits are associated with gastrointestinal diseases and investigated the role of Mediterranean diet in the gastrointestinal track, there are not sufficient data about the role of specific functional foods categories in gastrointestinal diseases. The decision of conducting this study is related to the fact that there has been only a limited number of studies about the possible effects of specific functional foods and bioactive compounds, which synergistically compose the Mediterranean diet, in gastrointestinal diseases.

Recent studies conclude that some categories of functional foods such as probiotics, prebiotics and herbs have a possible positive effect in the gastrointestinal system. Nowadays, the food industry is constantly developing novel functional foods containing live beneficial microorganisms [18]. Probiotics have probably a positive effect in inflammatory bowel disease attenuation, while prebiotics contain fibers which may prevent the development of inflammatory bowel disease [19,20,21]. Triangles, elm, and pie plant are herbs with possible positive effects in inflammatory bowel disease, while mastic may reduce heartburn which is a symptom of gastroesophageal reflux disease [22]. In a pilot study from Bundy et al. (2004), turmeric extract improved the irritable bowel syndrome symptomology in otherwise healthy adults [23]. Blueberries may reduce diarrhea which is a common symptom of gastrointestinal diseases [24]. According to a double-blind trial, *Aloe vera* reduced the problem of chronic constipation [25], while a pilot randomized positive-controlled trial showed that *A. vera* syrup had a positive effect in gastroesophageal reflux disease [26].

The possible effect of functional foods on the gastrointestinal system and, in general, on health promotion has been underlined for balanced diets such as the Mediterranean diet [12]. This diet includes high consumption of vegetables, fruits, olive oil, cereals, fishes and low consumption of meat and saturated fats. The Mediterranean diet has been studied as a possible effective factor in degenerative diseases’ prevention, such as cardiovascular and gastrointestinal diseases. Epidemiological data support that the possible beneficial effects of the Mediterranean diet are not the result of its individual components but are attributed to potential synergistic actions of its multiple bioactive compounds from various natural functional foods [17]. The benefits of the Mediterranean diet for the gastrointestinal tract have been previously underlined. In a recent case–control study in Italy, Zito et al. (2016) concluded that low adherence to the Mediterranean diet may trigger functional gastrointestinal symptoms, mainly in younger subjects with irritable bowel syndrome and functional dyspepsia [27]. In a recent study, Bhesania and Cresci (2017) reviewed the beneficial properties of the Mediterranean diet in patients with irritable bowel syndrome and concluded that the foods this diet is composed of may be useful in the specific patient population. Moreover, they underlined the need for promotion of nutritional education in order to improve the adherence to this diet [28]. In addition, bioactive compounds of the Mediterranean diet may attenuate chronic inflammation via a possible effect on C-reactive protein (HS-CRP), interleukin-6 (IL-6), and tumor necrosis factor alpha (TNF-α) [29]. In a recent review, the authors concluded that inflammation-induced platelet-activating factor (PAF) leads to the onset of cardiovascular diseases (CVD) rather than the increase of serum cholesterol levels [30].

The aim of the present study was to investigate the hypothesis that increased functional foods consumption and Mediterranean diet adherence have a positive effect in gastrointestinal diseases. The hypothesis was tested by a retrospective, randomized, case–control study in a sample of the Greek population.

## 2. Materials and Methods

### 2.1. Subjects

The study protocol was approved by the Hospitals’ Ethic Committee (approval code: 155, approval date: 12 April 2017), and the study was performed in accordance with the Declaration of Helsinki of 1975, revised in 2013. All participants signed an informed consent form and were informed about the prime target of the study, the confidentiality of data, and the voluntary nature of participation.

In total, 289 participants, 127 men and 162 women, aged 20–75 years were recruited from the Greek general hospitals G. H. Gennimata of Athens, G. H. Konstantopoulio of Athens, and G. H. Ippokrateio of Thessaloniki, either from the Department of Gastrointestinal Diseases or from other departments. Among the subjects, 142 were recruited from the hospital departments of gastrointestinal diseases and were diagnosed with ulcerative colitis, Crohn’s disease, irritable bowel syndrome, or gastroesophageal reflux disease. The patients visited or were monitored in the hospitals and had no other chronic diseases, such as cardiovascular diseases, diabetes mellitus, cancer, or metabolic syndrome. The other 147 participants were also recruited from the same hospitals and were healthy adults with no chronic inflammation and no chronic disease. They had visited the hospitals for routine medical tests. All participants were initially screened by using a medical history questionnaire.

### 2.2. Study Design

The study was a two-arm parallel, case–control study. Initially, 153 patients (cases) and 153 healthy (controls) were enrolled in this study. After screening the volunteers, 142 cases and 147 controls finally participated in the study, following the inclusion criteria. When a patient was enrolled in the study, a control also was enrolled, by simulation according to gender. The study started on 12 April 2017 and finished on 8 June 2017. During the study, the patients continued taking their medication related to gastrointestinal diseases. Both patients and healthy volunteers were visited by a nutritionist and completed two questionnaires, one for assessing their nutritional patterns and functional food consumption and the other for assessing the quality of their diet (adherence to the Mediterranean diet). The patients were asked to recall their nutritional habits in the 2–3 years before the disease diagnosis, while healthy volunteers recalled their nutritional habits in the 2–3 years before the enrolment in the study.

### 2.3. Questionnaires

The first questionnaire was a food frequency questionnaire (FFQ) based on the NHANES study FFQ after some modifications in order to include more natural foods. The questionnaire included four parts. The first contained questions about gender, age, occupation, and marital status. The second part contained 10 questions concerning the general and nutritional background of the participants, smoking, water abstraction, and daily meals, as well as the use of medication. In the third and fourth parts, the frequency of functional food categories–groups and specific functional foods and kinds of consumption were investigated. The possible answers were “every day”, “1–2 times a week”, “3–4 times a month”, “1–2 times a month”, and “never” [31].

The second questionnaire was a 14-item Mediterranean Diet Assessment Tool, designed by Martinez-Gonzalez et al. (2012), consisting of 14 questions about dietary habits in order to evaluate the quality of diet and especially the adherence to the Mediterranean diet. The possible answers were “yes or no”, codified as one point if the participant answered yes and 0 if the participant answered no. MedDiet Index <7 shows the lowest adherence to the Mediterranean diet, while 8–9 or >10 indicates the highest [32].

### 2.4. Statistical Analysis

The statistical analysis was performed by SPSS VER.22 (Statistical Package for Social Sciences, IBM, Armonk, NY, USA) using Kolmogorov–Smirov, chi-square test, non-parameter check U test, Spearman test, Hosmor–Lemeshons criterion, and models of logarithmic regression. Associations between categorical variables were examined using the chi -quare test. Differences were considered significant at *p* < 0.05, coefficient interval 95%.

## 3. Results

Case and controls showed no statistically significant differences relative to the gender (*p* = 0.184). Statistically significant differences were found between cases and controls about age, weight, body mass index, job, level of education, and level of physical activity (*p* < 0.05). The majority of patients were aged 51–65 years, while the majority of healthy people were aged 36–50 years. Cases had lower mean weight than controls, lower levels of physical activity, and higher body mass indexes (BMI) (Table 1).

Statistically significant differences were found between cases and controls in all categories of functional foods (*p* < 0.05). The percent of controls that consumed probiotics was 20 times higher than the relative percent of cases. Furthermore, controls consumed in general more categories of functional foods (prebiotics, foods enriched with vitamins and minerals, low-fat foods, and herb drinks) than cases (*p* < 0.05) (Table 2).

Statistically significant differences were found between patients and healthy volunteers in several kinds of functional foods (*p* < 0.05). Particularly, cases consumed in general less kinds of functional foods than controls, with the exception of cocoa which was consumed more by cases than controls (*p* < 0.05). Hippophae, Spirulina, and Royal jelly consumption was very low. So, although *p* < 0.05 the results for these foods were not considered significant (Table 3).

According to MedDiet Index, there were statistically significant differences between cases and controls. Specifically, controls showed an increased Index compared to cases 2–3 years before the study. The value of MedDiet Index was 9.08 for controls and 6.21 for cases (*p* < 0,05). Therefore, the healthy subjects had a better quality of nutrition than the patients in the years before disease diagnosis.

In Table 4, multifactorial analysis results are presented in order to evaluate the relationship between scores for functional food kinds and categories and the likelihood of gastrointestinal diseases. This analysis was performed using three models. Gender, age, body mass index, occupation, level of physical activity, and smoking habits of the participants were included in the three models. Furthermore, the functional food categories index was also added in model 2, and the functional food kinds index was added in model 3.

Model 1: The increase of body mass index and smoking were aggravating factors for gastrointestinal diseases. Someone who was a smoker had a higher probability (specifically, 4.17 times) to develop gastrointestinal diseases than someone who was not a smoker. In regard to the level of physical activity, the participants with high level of physical activity (3–4 times per week) had 72% lower probability to develop gastrointestinal disease than the participants with very low levels of physical activity (1–2 times per week). Age and gender did not play a statistically significant role.

Model 2: Only the index related to the categories of functional food showed a statistically significant effect. Especially, all categories of functional foods were protective factors for gastrointestinal diseases (OR = 0.69).

Model 3: Only the index related to the kinds of functional food showed a statistically significant role. More specifically, the kinds of functional foods were protective factors for gastrointestinal diseases (OR = 0.90) (Table 4).

In Table 5, further analyses were made to evaluate the relationship between the scores for functional food categories and kinds and the likelihood of gastrointestinal diseases. This relationship was investigated by three models. Gender, age, medication, smoking and other diseases were included in the three models. Also, functional food categories, and kinds indexes were added in model 3.

Model 1: Smoking, dietary supplements, and medication were aggravating factors for gastrointestinal diseases. Participants who were smokers were 3.85 times more likely to develop a gastrointestinal disease than participants who were non-smokers. According to dietary supplements, participants who occasionally consumed dietary supplements were 6.18 times more likely to develop a gastrointestinal disease than participants who did not consume dietary supplements, whereas participants who were taking drugs were 12.56 times more likely to develop a gastrointestinal disease than participants who were not taking drugs.

Model 2: Participants who occasionally consumed dietary supplements were 43.16 times more likely to develop a gastrointestinal disease than participants who did not consume dietary supplements. Medication was an aggravating factor, because participants who were taking drugs were 12.37 times more likely to develop a gastrointestinal disease than participants who were not taking drugs. Age, gender, smoking, and suffering from other diseases did not show a statistically significant effect. The increase of the index related to the consumption of functional foods categories was a protective factor for gastrointestinal diseases (OR = 0.64).

Model 3: Participants with >65 years old were 7 times more likely to develop gastrointestinal diseases than participants aged 20–35 years. Participants who consumed dietary supplements were 96.44 times more likely to develop gastrointestinal diseases than participants who did not consume dietary supplements. Medication was an aggravating factor because participants who were taking drugs were 18.97 times more likely to develop gastrointestinal diseases than participants who were not taking drugs. Also, participants who suffered from other diseases were 82 times less likely to develop gastrointestinal diseases than participants who did not suffer from other diseases. The increase of the functional foods kind index was a protective factor for gastrointestinal diseases (OR = 0.86) (Table 5).

In Table 6, multifactorial analysis results are presented in order to evaluate the relationship between the scores for functional food categories, functional food kinds, dietary habits based on the Mediterranean diet and gastrointestinal diseases. The results of this analysis showed that age and gender did not play a statistically significant role. The increase of Mediterranean diet adherence and functional foods consumption index was a protective factor for gastrointestinal diseases appearance (OR = 0.70, 0.72, and 0.92 for MedDiet Index, functional foods category, and functional foods kind, respectively) (Table 6).

## 4. Discussion

The basic finding of this study is that patients with gastrointestinal diseases consumed less functional foods 2–3 years before disease diagnosis, especially probiotics, prebiotics, and herbs and plant foods rich in antioxidants than controls in the last 2–3 years prior to the study. Moreover, controls showed a higher Mediterranean diet Index than cases in this 2–3 years period, indicating that healthy subjects had a better quality of nutrition than patients. Thus, the study indicates a possible protective function of the consumption of specific functional foods and the adoption of the Mediterranean diet with respect to gastrointestinal diseases’ appearance. Another important finding is that smoking, dietary supplements, medication, and the increase of BMI were aggravating factors for gastrointestinal diseases. Physical activity did not have a statistically significant role in gastrointestinal diseases with the exception of high levels of physical activity showing a protective effect. Participants who were >65 years old had an increased risk of developing gastrointestinal diseases.

Although many studies have indicated that dietary habits and specific foods consumption are associated with the appearance or prevention of gastrointestinal diseases, there are few studies that investigate if specific functional foods categories and kinds have a positive effect counteracting the development of those diseases.

Research data suggest that the possible effect of functional foods consumption should be connected with the adoption of a balanced diet with high quality, such as the Mediterranean diet. The adherence to this diet was associated in the present study with a decreased risk of gastrointestinal diseases’ appearance. In a relative population-based case–control study of inflammatory bowel disease and dietary habits in Stockholm, which obtained retrospective information about food intake 5 years previously for 152 cases with Crohn’s disease, 145 cases with ulcerative colitis, and 305 controls, the relative risk of Crohn’s disease was increased for subjects who a had high intake of sucrose and was decreased for subjects who had a high intake of fiber. The relative risk associated with the consumption of fast foods at least two times a week was estimated at 3.4 for Crohn’s disease and 3.9 for ulcerative colitis [33]. The results of this study are associated with the basic conclusion of the present study that the adoption of a balanced diet may prevent gastrointestinal diseases. The same conclusion was reached in a recent epidemiological study in Italy in 2016, which concluded that increased adherence to the Mediterranean diet may prevent gastrointestinal symptoms [26].

In our study, fiber consumption had a positive effect in the prevention of gastrointestinal diseases. The same result was reported in another study which concluded that dietary fiber has many health-promoting effects on the gastrointestinal tract. Fiber maintains normal gut function and promotes regularity. Foods rich in soluble fiber, such as oat products, may delay gastric emptying and enhance satiety [34].

Another important result of our study was that probiotics consumption may protect from gastrointestinal diseases, giving the fact that the patients consumed less functional foods 2–3 years before disease diagnosis than healthy people. Probiotic bacteria may inhibit pathogens by various mechanisms, such as competitive exclusion, competition for substrates and limiting resources, synthesis of antimicrobial substances (organic acids, hydrogen peroxide, bacteriocins, etc.), inhibition of toxin expression from pathogens, etc., [35]. A systematic review and meta-analysis of randomized controlled trials, with 1182 patients, about the effect of probiotics on functional constipation in adults concluded that probiotics significantly reduced whole gut transit time by 12.4 h and increased stool frequency by 1.3 bowel movements/wk, (*Bifidobacterium lactis*), while improving stool consistency (*B. lactis*). There are several potential mechanisms of action by which probiotics may benefit functional constipation. First, probiotics may modify the gastrointestinal microbiota, which is known to be altered in constipation. Second, probiotic metabolites may alter gut function, including sensation and motility. Third, some probiotics increase the production of lactate and short-chain fatty acids reducing luminal pH, which, as some researchers have proposed, may enhance colonic peristalsis and shorten whole gut transit time [36]. Another systematic review and meta-analysis of six randomized controlled trials, showed that probiotics increased stool frequency and had beneficial effects in Asian children [37]. The results of these studies are in accordance with the results of our case–control study showing that probiotics were protective for gastrointestinal diseases.

In our study, functional foods rich in antioxidants, such as berries and herbs, appeared to be associated with a decreased risk of gastrointestinal diseases appearance. A double-blind clinical trial with 38 patients with symptomatic duodenal ulcer concluded that mastic, 1g daily for 2 weeks, has an ulcer-healing effect [38]. This study is in accordance with our study which indicated that mastic had a positive effect in gastrointestinal diseases. Antioxidant compounds in various natural functional foods, such as polyphenols, have been studied as bioactive compounds with possible beneficial effects due to their free radical scavenging activity or possible effects on metabolic pathways and gene expression [39,40].

The present study has some limitations. There are limitations such as revocation error and selection error due to the case–control design of the research. Accurate and detailed data were gathered to determine the revocation error. Also, conjunctive factors were not included in this study [41]. Moreover, cases and controls were similar only in relation to gender percent and not for other factors as age, etc. Furthermore, this is a retrospective study, and thus the results should be further confirmed by large prospective studies. The study sample was limited and included participants that were hospitalized in three specific hospitals in central and northern Greece. Larger epidemiological studies with samples from other areas of the country, urban, rural, and island regions are essential in order to reach more reliable conclusions regarding the Greek population.

## 5. Conclusions

The study indicates a possible protective association between the consumption of functional foods and the adoption of the Mediterranean diet on one hand and the possibility of gastrointestinal diseases appearance on the other. Further research should be conducted, especially large clinical and epidemiological studies, in order to further investigate the role of nutrition and bioactive compounds in gastrointestinal diseases and to promote the consumption of functional foods.

## Figures and Tables

**Table 1:** Cases–controls comparisons in relation to demographic characteristics.

Demographic Characteristics		Cases	Controls	*p*
**Gender**	**Men**	47.9%	40.1%	0.184
	**Women**	52.1%	59.9%	
**Age**	**20–35 Years Old**	19%	25.9%	0.004
	**36–50 Years Old**	23.9%	36.1%	
	**51–65 Years Old**	33.8%	27.9%	
	**>65 Years Old**	23.2%	10.2%	
**Level of Physical Activity**	**Very Low (1–2/month)**	13.4%	6.1%	0.014
	**Low (3–4/month)**	16.9%	9.5%	
	**Moderate (1–2/week)**	40.1%	44.2%	
	**High (3–4/week)**	15.5%	27.9%	
**Weight in kg**		75.43 ± 16,41	69.12 ± 12.36	0.001
**Height in m**		1.69 ± 0.09	1.69 ± 0.08	0.915
**Body Mass Index**		26.43 ± 5.35	24.12 ± 3.49	<0.001

*n* = 142 for cases and 147 for controls.

**Table 2:** Cases–controls comparisons in relation to the consumption frequency of functional foods categories.

Consumption/Functional Foods Categories	Never		Very Rarely (1–2/Month)		Rarely (3–4/Month)		Frequently (1–2/Week)		Very Frequently (3–4/Week)		Every Day		*p*
	Cases	Controls	Cases	Controls	Cases	Controls	Cases	Controls	Cases	Controls	Cases	Controls	
**Probiotics**	66.9%	13.6%	21.1%	17.7%	9.2%	15.6%	2.1%	40.1%	0.7%	6.8%	0%	6.1%	<0.001
**Prebiotics**	56.3%	6.1%	15.5%	8.8%	19%	11.6%	6.3%	37.4%	2.8%	16.3%	0%	19.7%	<0.001
**Foods Enriched with Vitamins and Minerals**	63.4%	12.2%	19%	20.4%	12.7%	19%	2.8%	22.4%	1.4%	12.9%	0.7%	12.9%	<0.001
**Low-Fat Foods**	64.1%	10.2%	14.1%	15%	14.1%	15.6%	5.6%	32.7%	2.1%	17%	0%	9.5%	<0.001
**Herbs Drinks**	26.8%	4.1%	22.5%	9.5%	35.2%	17%	13.4%	41.5%	1.4%	17.7%	0.7%	10.2%	<0.001

*n* = 142 for cases and 147 for controls.

**Table 3:** Cases–controls comparisons in relation to the consumption frequency of functional foods kinds.

Consumption/Functional Foods Kinds	Never		Very Rarely (1–2/Month)		Rarely (3–4/Month)		Frequently (1–2/Week)		Very Frequently (3–4/Week)		Every Day		*p*
	Cases	Controls	Cases	Controls	Cases	Controls	Cases	Controls	Cases	Controls	Cases	Controls	
**Goji Berry Cranberry Berries**	70.4%	19.7%	14.1%	17.7%	12.7%	23.8%	2.8%	30.6%	0%	6.1%	0%	2%	<0.001
**Tea**	28.9%	4.1%	27.5%	17%	32.4%	23.8%	9.9%	39.5%	0.7%	10.2%	0.7%	5.4%	<0.001
**Broccoli Crumbly Vegetables**	26.1%	2%	15.5%	6.1%	33.1%	9.5%	20.4%	53.7%	4.9%	23.8%	0%	4.8%	<0.001
Hippophae *	95.1%	81%	2.8%	5.4%	2.1%	4.8%	0%	4.8%	0%	3.4%	0%	0.7%	0.001
Spirulina *	95.1%	89.1%	2.8%	5.4%	2.1%	2%	0%	2.7%	0%	0%	0%	0.7%	0.142
Royal jelly *	94.4%	86.4%	2.8%	5.4%	2.8%	2.7%	0%	1.4%	0%	1.4%	0%	2.7%	0.080
**Mountain tea**	29.6%	7.5%	26.1%	14.3%	31%	20.4%	11.3%	38.8%	0.7%	15.6%	1.4%	3.4%	<0.001
**Linseed**	92.3%	59.9%	6.3%	12.9%	0.7%	10.2%	0.7%	7.5%	0%	4.1%	0%	5.4%	<0.001
**Pomegranate**	43%	15.6%	31%	14.3%	22.5%	23.1%	3.5%	34.7%	0%	11.6%	0%	0.7%	<0.001
**Oats**	78.9%	25.2%	7%	12.2%	5.6%	19%	3.5%	21.8%	1.4%	10.2%	3.5%	11.6%	<0.001
**Kefir**	96.5%	56.5%	2.8%	14.3%	0.7%	14.3%	0%	12.2%	0%	1.4%	0%	1.4%	<0.001
**Ginger**	90.1%	55.1%	5.6%	15.6%	4.2%	12.2%	0%	11.6%	0%	4.1%	0%	1.4%	<0.001
**Cocoa**	47.9%	33.3%	12%	19%	19.7%	29.3%	12%	11.6%	4.2%	6.1%	4.2%	0.7%	0.023
**Raisin**	40.1%	7.5%	32.4%	19%	21.8%	21.1%	4.9%	32%	0%	12.9%	0.7%	7.5%	<0.001
**Soya**	91.5%	83%	6.3%	6.8%	1.4%	7.5%	0.7%	2%	0%	0%	0%	0.7%	0.049
**Sage**	45.8%	43.5%	21.1%	17.7%	23.9%	21.1%	7.7%	12.2%	0.7%	4.1%	0.7%	1.4%	0.317
**Gum**	71.8%	34%	14.8%	23.1%	9.2%	15.6%	2.1%	19.7%	2.1%	5.4%	0%	2%	<0.001
**Thyme**	67.6%	20.4%	14.8%	12.9%	15.5%	19%	2.1%	32.7%	0%	11.6%	0%	3.4%	<0.001
**Turmeric**	90.8%	44.9%	4.2%	15%	4.2%	12.9%	0.7%	16.3%	0%	4.8%	0%	6.1%	<0.001
**Other**	100%	95.2%	0%	1.4%	0%	0.7%	0%	0.7%	0%	1.4%	0%	0.7%	0.226

*n* = 142 for cases and 147 for controls. * Hippophae, Spirulina, and Royal jelly consumption was very low; so, although *p* < 0.05, the results were not considered significant.

**Table 4:** Multifactorial analysis results to evaluate the relationship between the scores for functional food categories and for functional food kinds and the likelihood of gastrointestinal diseases, based on age, occupation, and physical activity.

Factors	Model 1	Model 2	Model 3
**Women vs. Men**	0.66 (0.37, 1.17)	1.35 (0.56, 3.25)	1.11(0.45, 2.75)
**Age**			
**20–35 Years Old**	1	1	1
**36–50 Years Old**	0.71 (0.29, 1.76)	1.23 (0.34, 4.49)	1.34 (0.36, 5.03)
**51–65 Years Old**	0.70 (0.27, 1.78)	1.16 (0.29, 4.59)	1.65 (0.39, 7.04)
**>65 Years Old**	0.59 (0.16, 2.20)	0.33 (0.04, 3.09)	0.37 (0.03, 4.12)
	1.09 (1.02, 1.17)	1.10 (0.99, 1.21)	1.10 (0.99, 1.22)
**Employment unemployed**	1	1	1
**Employed**	0.22 (0.09, 0.55)	0.12 (0.03, 0.47)	0.12 (0.03, 0.48)
**Student**	0.13 (0.04, 0.48)	0.11 (0.01, 0.78)	0.12 (0.02, 0.90)
**Retired**	1.52 (0.49, 4.75)	1.50 (0.22, 10.44)	1.66 (0.20, 13.84)
**Physical Activity Level**			
**Very Low (1–2 Times/Month)**	1	1	1
**Low (3–4 Times/Month)**	1.06 (0.32, 3.46)	1.60 (0.30, 8.48)	1.50 (0.28, 7.89)
**Moderate (1–2 Times/Week)**	0.46 (0.17, 1.27)	1.21 (0.32, 4.64)	1.31 (0.32, 5.28)
**High (3–4 Times/Week)**	0.28 (0.10, 0.85)	1.14 (0.24, 5.48)	1.11 (0.21, 5.78)
**Very high (Every Day)**	0.62 (0.19, 1.98)	2.51 (0.48, 13.18)	1.90 (0.35,10.23)
**Smokers Against Non-Smokers**	4.17 (2.26, 7.71)	1.64 (0.67, 3.99)	1.35 (0.54, 3.35)
**Categories of Functional Food Score**	-	0.61 (0.53, 0.69)	0.69 (0.60, 0.80)
**Kinds of Functional Food Score**	-	-	0.90 (0.84, 0.97)

Values of Odds Ratio (OR) with Coefficient Interval (CI) in parenthesis. *n* = 142 cases and *n* = 147 controls.

**Table 5:** Multifactorial analysis results for the relationship between the scores for functional food categories and for functional food kinds and the likelihood of gastrointestinal diseases based on gender, smoking, medication, and supplements habits.

Factors	Model 1	Model 2	Model 3
**Women vs. Men**	0.72 (0.39, 1.33)	1.39 (0.52, 3.68)	1.66 (0.59, 4.68)
**Age**			
**20–35 Years Old**	1	1	1
**36–50 Years Old**	1.33 (0.56, 3.15)	1.75 (0.46, 6.65)	2.17 (0.51, 9.19)
**51–65 Years Old**	2.28 (0.95, 5.47)	2.92 (0.67, 12.69)	4.99 (0.94, 26.59)
**>65 Years Old**	5.04 (1.70, 14.99)	4.50 (0.80, 25.26)	7.11 (1.13, 44.80)
**Smokers Against Non-Smokers**	3.85 (1.98, 7.49)	1.54 (0.54, 4.42)	1.08 (0.36, 3.24)
**Dietary Supplements**			
**Never**	1	1	1
**Every day**	2.99 (0.96, 9.30)	3.59 (0.55, 23.51)	4.30 (0.56, 32.78)
**Weekly**	2.65 (0.35, 20.30)	1.04 (0.05, 23.15)	1.45 (0.07, 28.42)
**Occasionally**	6.18 (2.70, 14.15)	43.16 (9.15, 203.55)	96.44 (16.33, 569.64)
**Medication (Yes Against No)**	12.56 (5.97, 26.42)	12.37 (3.90, 39.18)	18.97 (5.05, 71.18)
**Other Diseases**	0.41 (0.17, 0.95)	0.29 (0.08, 1.09)	0.18 (0.04, 0.77)
**Categories 0f Functional Food Score**	-	0.57 (0.49, 0.66)	0.64 (0.54, 0.76)
**Kinds of Functional Food Score**	-	-	0.86 (0.79, 0.93)

Values of Odds Ratio (OR) with Coefficient Interval (CI) in parenthesis. *n* = 142 cases and *n* = 147 controls.

**Table 6:** Multifactorial analysis results for the evaluation of the relationship between the scores for functional food categories, functional food kinds, and dietary habits and gastrointestinal diseases, based on Mediterranean diet adherence.

Factors	Odds Ratio	95% CI
**Women vs. Men**	1.38	0.60, 3.21
**Age**		
**20–35 Years Old**	1	1
**36–50 Years Old**	1.31	0.44, 3.88
**51–65 Years Old**	3.14	0.98, 10.10
**>65 Years Old**	3.85	0.94, 15.72
**Med Diet Score**	0.70	0.56, 0.88
**Categories of Functional Food Score**	0.72	0.63, 0.82
**Kinds of Functional Food Score**	0.92	0.87, 0.98

*n* = 142 cases and *n* = 147 controls.
